# Self-Care Management, Patient Education, and Nursing Support in Patients with Diabetes and Hypertension

**DOI:** 10.31661/gmj.v13i.3166

**Published:** 2024-02-08

**Authors:** Amirabbas Rostami, Hamid Reza Sabet, Eftekhar Azarm, Haleh Kangari, Zahra Elihaei, Babak Khodadoustan Shahraki, Parisa Hosseini Koukamari

**Affiliations:** ^1^ Department of Internal Medicine, School of Medicine , Isfahan University of Medical Sciences , Isfahan, Iran; ^2^ Medical Journalism Department, School of Paramedical Sciences, Shiraz University of Medical Sciences, Shiraz, Iran; ^3^ School of Medicine, Isfahan University of Medical Sciences, Isfahan, Iran; ^4^ Department of optometry, School of Rehabilitation, Shahid Beheshti University of Medical Sciences, Tehran, Iran; ^5^ Department of Nursing, School of Nursing and Midwifery, Ahvaz Jundishapur University of Medical Sciences, Ahvaz, Iran; ^6^ Isfahan university of medical sciences, Isfahan, Iran; ^7^ Department of Public Health, Student Research Committee, Saveh University of Medical Sciences, Saveh, Iran

**Keywords:** Self-Care, Diabetes, Hypertension, Education, Lifestyle

## Abstract

Diabetes and related complications such as hypertension are considered major public health issues throughout the world as they are the most important causes of death and disability and cause huge economic implications for both patients and the public health system. Sufficient management of these complications along with novel patient education and self-care strategies are documented as ideal approaches able to postpone the onset of morbidity and disability and thereby delay the onset of preventable costs. However, contradictions regarding the efficacy of the studied self-care strategies are mentioned, thereby the current study aimed to review self-care strategies as a preventive approach to disease management and improving clinical outcomes in patients with diabetes and hypertension. The findings revealed that self-care through patient education with nursing support can improve medication adherence and lifestyle. However, clinical outcomes have revealed relative inconsistencies that require further studies.

## Introduction

Chronic non-communicable diseases are considered a major public health issue throughout the world, among which diabetes and cardiovascular disorders (CVDs) are described as the most notable diseases [1][2]. Although it was previously estimated that the number of patients with diabetes around the world will increase by approximately 60 percent from 382 million to 592 million between 2013 and 2035 [3], the International Diabetes Federation (IDF) has recently estimated that the number of diabetic patients projected to rise to 643 million by 2030 and 783 million by 2045 [4]. Also, IDF in 2023 estimated that 537 million individuals had diabetes worldwide, and 75 percent of them live in low- and middle-income countries [4]. Moreover, the study of the global burden of disease, risk factors, and disabilities has confirmed that CVDs are the leading cause of mortality while hypertension, a major cause of premature death worldwide, is described as the globally major risk factor [5]. In fact, the World Health Organisation estimated that 1.28 billion people suffer from hypertension worldwide, and two-thirds of these individuals live in low and middle-income regions [5].

Hence, the rising burden of the mentioned diseases is challenging public health globally. It is widely documented that diabetes and related complications such as CVDs and hypertension cause huge economic implications for both patients and the public health system. The listed economic implications include direct huge costs incurred by both individuals and their families as well as by governmental agencies responsible for the management of the disease. Moreover, indirect costs result from a loss of wages and/or decreased productivity at work because of a person’s disability.

Notably, chronic diseases such as diabetes and hypertension are able to impose a vast amount of direct and indirect costs in both high-income nations (e.g. the United States of America) and middle- and low-income nations (e.g. India and a number of African countries) [6][7][8]. Moreover, the admission and subsequent complications of diabetes could remarkably increase the mentioned costs [9][10]. There is a plethora of evidence demonstrating that the appropriate management of diabetes and risk factors such as hypertension could significantly postpone the onset of morbidity and disability and thereby delay the onset of preventable costs [11][12].

Therefore, it is imperative that health systems are geared towards controlling patients with diabetes and other risk factors appropriately in order to detect the disease early, delay complications, and prevent hospital admissions [13]. Primary health care provides tremendous opportunities regarding the care of chronic non-communicable complications resulting in cost-saving [13]. Self-care, for example, is described as a fundamental element of treatment for patients with a chronic condition. Indeed, the self-care of diabetic patients is a major focus of many interventions and a plethora of research exists describing different types of self-care interventions [14]. It should be stated that numerous sources have noted that proper disease management and novel patient education and self-care strategies may prevent the consequences of these complications and significantly reduce the huge public costs, disability, and mortality. Nevertheless, there are some kind of contradictions regarding the efficacy of the studied self-care methods. As a result, the current study aimed to review self-care strategies as a preventive /confrontational approach to managing the disease and improving clinical outcomes in patients with diabetes and hypertension.

The Standard for Organization of Health Care, Patient Support, and Self-care

The chronic care model (CCM) is described as a comprehensive standard of care for the organization of healthcare services for chronic diseases that emerged in the 1990s. The concept of CCM has been refined over the past years and is made on evidence that multicomponent structured care could be followed by improved outcomes for chronic non-communicable diseases [15]. Regarding the standard of medical care for diabetes, CCM is recommended by the American Diabetes Association for the organization of care by healthcare networks [16]. CCM defines and restructures care to cause partnerships between communities and health systems. Chronic care occurs within three major areas including the entire community, the health care system, and the provider organization that could be an integrated big delivery system or just a small clinic/primary care practice [13]. It is documented that the CCM recognizes the following six essential elements:

1. Health system for the organization of health care by providing leadership for securing resources and reducing, and removing barriers to care;

2. Self-management support by facilitating skill-based learning and patient empowerment;

3. Decision support by guiding for implementing evidence-based care;

4. Delivery system design contributing to coordinating care processes;

5. Clinical information systems track progress through reporting outcomes to patients and providers;

6. Community resources and policies sustaining care by the application of community-based resources and public health policy.

The ultimate aim of CCM is to envision an informed and activated patient interacting with a prepared and proactive practice team that results in high-quality, satisfying encounters, and finally improved clinical outcomes [17][18]. Since the burden of chronic diseases is increasing, the majority of chronic care is delivered in the primary care setting, thereby a considerable amount of time primary care physicians spend on confronting chronic diseases and supporting patients; as a result, global primary care practices require to be organized in order to provide high-quality care and educate patients.

Patient support groups are considered a component of CCM and a high-potential self-management strategy that is able to improve the care for chronic diseases such as diabetes and hypertension. It is evidenced that patient-led support teams may represent an ideological shift away from the perception of uninformed patients as ‘passive’ recipients of treatment to educated empowered individuals who are partners in the active effective management of their own condition [19][20]. Peer support has been demonstrated to improve healthy behaviors in diabetic patients in undeveloped countries [21].

Support groups are responsible for a wide range of roles from improving treatment adherence, self-monitoring, reporting of disease, and providing emotional support to induce behavioral and lifestyle adjustments [21][22]. As a result, support groups have produced considerable interest as a strategy for reaching low-income patients. Due to the scarcity of public and human resources in low- and middle-income countries for the management of patients with diabetes and hypertension, innovative and self-sustaining/self-care approaches are pivotally required for the improvement of the care of chronic disease patients [23]. Self-care, as mentioned earlier, is considered a principal and integral component of treatment in chronic disease and patients who engage in self-care represent a significant improvement in their clinical outcomes which is accompanied by a higher quality of life, lower rate of hospital admission, and longer survival [24]. In fact, self-care has exponentially grown over the past decade mainly because of the availability of self-report instruments and applicable theories [25][26][27][28]. Although multiple studies have described a variety of self-care interventions specific to different pathological conditions [24][29], healthcare is compartmentalized into specialties and subspecialties that usually prevent the delivery of information between different disciplines [14]. Self-care for chronic diseases has been theoretically described as the process of maintaining health through health-promoting practices and managing disease [30], thus self-care encompasses a variety of behaviors including both general and disease-specific, in which individuals suffering from a chronic disease engage to maintain their physical and emotional stability.

The mentioned behaviors such as adherence to medications, managing stress, ensuring appropriate and sufficient sleep, and physical activity are considered self-care maintenance, while sef-care monitoring is referred to as the process of behavior monitoring for alterations in disease symptoms [31]. It is documented that self-care interventions are heterogeneous for specific patient groups in targeted self-care behaviors such as lifestyle and medication adherence, the intensity of intervention, and the obtained outcomes which in turn has mde it difficult to reach evidenced recommendations for clinical practices. Hence, self-care interventions for each specific disease and patient group must be reviewd and interventions and outcomed must be assessed to understand the efficacy of studied approaches [32][33]. In the following, the current study will review and discuss self-care interventions for patients with diabetes and hypertension.

Self-care Approaches Could Improve Adherence to Medications

It is widely established that medication nonadherence is a common, costly, and underdiagnosed occurrence in patients with chronic disease. It is accepted that adherence to medications is pivotally necessary for the improvement of outcomes and decrement of healthcare costs in chronic diseases such as diabetes [34], and medication nonadherence results in poor health outcomes, increased risk of hospital admission, and higher rates of mortality [35][36]. As the treatment regimen for diabetic patients is complex, it is difficult to adherent to diabetic medication and it has been demonstrated that medication adherence in patients with diabetes is worse than in patients with other chronic diseases [37].

The improvement of medication adherence is believed to be a high efficient strategy than altering the treatment regimen [38] since self-care protocols seek to provide an efficient approach to improve medication adherence. A recent study by Valdenor et al. has revealed that in patients with at least one chronic cardiometabolic disease including diabetes, hypertension, atrial fibrillation, or heart failure who were taking medications for their own disease, primary care physicians were not able to confront medication nonadherence [39], hence self-care strategies such as educating patients about the importance of medication adherence and behavioral interventions should be emphasized.

Raj et al. in a pilot randomized controlled trial, involving 50 elderly inpatients of general medicine wards diagnosed with select non-communicable chronic diseases, such as diabetes and hypertension, investigated the impact of behavioral intervention on medication adherence [40]. The results of this open-label, single-center, parallel-arm controlled trial revealed that behavioral interventions including receiving the usual standard of care or the intervention comprising of patient diary to mark daily prescribed medication intake, systematic education, and periodic telephone reminders for both 3 (91.88% compared to 78.20% in controls) and 6 months (83.08% compared to 68.64% in controls) could be followed by a significant improvement in medication adherence [40].

In addition, a four-year, parallel, randomized controlled clinical trial involved 50 individuals to assess the impact of participating in self-management education in comparison with individual education on clinical and psychological variables in patients with diabetes and hypertension [41]. The outcomes revealed that an educational approach based on the multidisciplinary structured group is able to improve medication adherence and blood pressure in diabetic patients [41]. Concordantly, Parra et al. in a parallel randomized two-arm clinical trial involving 200 patients with hypertension and/or diabetes demonstrated that nursing intervention involving individual teaching in comparison with usual care is able to improve medication adherence significantly [42]. Due to the ubiquity of smartphones in individuals’ daily lives, several studies have considered a high potential for mobile health (mHealth) tools in order to facilitate and improve medication adherence in patients with chronic diseases [43]. In this regard, Sartori et al. conducted a controlled randomized clinical trial to investigate the efficacy of WhatsApp-based education intervention in the adherence to antidiabetic and antihypertensive medication in patients with hypertension and diabetes, which showed that education of patients by WhatsApp probably could function as a reinforcement to improve medication adherence, however, clinical outcomes were not discussed [44].

Similarly, a pilot randomized feasibility trial on black patients with diabetes and hypertension revealed that a tailored mHealth intervention is able to significantly improve medication adherence and clinical outcomes. Nevertheless, the results showed no remarkable difference when compared to an attention control intervention for medication adherence [45]. Therefore, one can conclude that self-care strategies based on educational interventions, whether using mHealth tools or other methods, can lead to adherence to prescribed medication regimens and, as a result, desired therapeutic outcomes.

Self-care Approaches to Improve the Lifestyle of Patients with Diabetes and Hypertension

It is widely suggested that patients with diabetes and hypertension must change their lifestyle including the reduction of weight, increment of physical activity, and development of behavior skills [45][46][39]. In fact, diabetic patients are referred to a structured intensive lifestyle intervention program to pursue 7% weight loss and a long-term goal of 150 minutes weekly of moderate-intensity physical activity. It has been demonstrated that intensive lifestyle intervention is able to postpone the onset of diabetes by approximately 4 years and decrease the overall incidence of diabetes by about 34% over ten years [47]. In addition, skill development and self-monitoring such as the careful tracking of weight and food quantity along with measurement of heart rate and blood pressure are listed as self-care skills required for lifestyle change [48]. Interestingly, Trento and colleagues have determined that self-care education is able to improve blood pressure in diabetic patients by improving medication adherence and lifestyle changes [41]. Along with that, a 6-month hospital-initiated and discharge/community nurses-coordinated transitional care intervention could cause a remarkable decrease in both systolic and diastolic blood pressures as well as a nonsignificant improvement in the levels of HbA1c, knowledge of hypertension and diabetes, medication adherence, quality of life, hospital readmission in elder patients with hypertension and diabetes [46].

Previous studies have investigated several lifestyle-based self-care approaches in patients with diabetes and hypertension. Wilkinson et al. have shown that a lifestyle intervention consisting of 10-hour time-restricted eating can significantly reduce weight, blood pressure, and atherogenic lipids in patients [49]. Meanwhile, home blood pressure monitoring along with lifestyle intervention on weight, blood pressure, and self-efficacy failed to improve blood pressure one year after delivery [50].

The evaluation of a package of risk-based medication and lifestyle interventions in a pragmatic cluster randomized controlled trial by Wei et al. has determined that they have no significant effect on CVD-related complications such as diabetes and blood pressure [51].

Furthermore, it is demonstrated that the addition of web-based behavioral support in order to improve exercise referral schemes in 450 people aged 16-74 years, with chronic complications such as obesity, hypertension, type 2 diabetes, etc., had only a weak nonsignificant indicative impact on long-term moderate and vigorous physical activity [52]. Therefore, it appears that self-care strategies and care support approaches to improve lifestyle have less impressive outcomes compared to medication adherence findings.

Self-care May Improve Clinical Outcomes in Patients with Hypertension and Diabetes

As mentioned earlier, several conducted studies did not investigate the clinical outcomes of self-care in diabetes and hypertension patients and also clarified contradictions regarding the effectiveness, especially in the case of lifestyle interventions. Therefore, in this part, the present study deals with the clinical findings and existing contradictions regarding the effectiveness of self-care strategies in the treatment of patients with diabetes and hypertension. It was previously described that 36 months of risk-based drug and lifestyle interventions in 13,385 patients with diabetes and hypertension significantly increased medication adherence and had a small effect on systolic blood pressure reduction. However, the mentioned intervention could not have any significant effect on the incidence rate of CVDs [51].

This is despite the fact that individual education of the patient by the nurse, increasing their awareness of the disease, and informing patients about how to do self-care represented desired outcomes. In fact, Li et al. conducted a study to investigate the nursing effect of self-care on 34 patients with diabetes and hypertension in comparison with patients who received routine nursing. The results demonstrated that the levels of fasting blood glucose, 2-hour postprandial glucose, and systolic and diastolic blood pressure in patients who received self-care nursing decreased significantly more than in the other group, suggesting that individualized self-care nursing and health education could effectively improve the psychological cognition and strengthen the control of blood pressure and blood sugar of patients with diabetes and hypertension [53]. Concordantly, Trento et al. revealed that self-care education is able to desirably improve blood pressure in patients with diabetes, presumably through improvement of medication adherence and lifestyle changes [41]. Although studies that emphasized nurse-led education have resulted in favorable findings, the investigation of other approaches in order to improve self-care has revealed contradictory and in some cases unpleasant results. The study of McManus et al., who investigated the effect of home and online digital intervention for the management of hypertension in primary care by the combination of self-monitoring of blood pressure with guided self-management, could be referred to as an example of the few studies that obtained positive results [54].

The mentioned unmasked randomized controlled trial conducted on 622 patients with poorly controlled and who had access to the internet showed that digital intervention, which provided feedback on blood pressure outcomes to patients and professionals along with optional lifestyle advice and motivational support, was able to remarkably reduce both systolic and diastolic blood pressure compared to patients who received routine care consisted of routine hypertension care and appointments and medication changes made at the discretion of the general physician [54].

Although these findings suggested a cost-effective and efficient approach to improving blood pressure in diabetic and elderly patients [54], other studies have reported negative results. A controlled randomized crossover study on 37 diabetic and hypertensive patients revealed that although integrative mobile health intervention (consisting of self-measurement of blood pressure and blood glucose levels, health education, lifestyle change, and medication monitoring and adherence) was able to elevate the input rate of taking medicine, no significant alteration in body weight, blood pressure, and HbA1c was achieved [55]. Accordingly, two other studies that sought to improve the clinical outcomes of patients through the use of mobile phones, one with nurse support and quality of life assessment [56] and the other to improve self-care health management [57], have reported the ineffectiveness of the interventions.

These contradictions regarding the effectiveness of self-care approaches in improving clinical outcomes of patients with diabetes and hypertension may be dependent on the type of approach. For example, in the evaluation of facilitators and barriers for improving self-management and patient support, it has been determined that voice messages are preferred over texts by both patients and primary health care providers to communicate trust and increase accessibility for patients with low levels of literacy, limited vision, and smartphone unavailability [58]. In addition, the lack of significant clinical outcomes can be attributed to the racial differences of the patients participating in the study, as Kobe et al. showed that a pharmacist-delivered telehealth intervention could be more effective in African-American patients than non-African Americans [47].

## Conclusion

The reviewed studies determined that self-care approaches along with health education and nursing support can effectively improve medication adherence in patients with diabetes and hypertension and to some extent change lifestyle for the benefit of health. However, there are inconsistencies regarding the improvement of clinical outcomes, which may be related to differences in approaches and racial diversity of participants. Therefore, determining the clinical effectiveness of these approaches and suggesting more ideal strategies requires further studies.

## Conflict of Interest

none declared.
